# Transfusion consent in Brazil: A nationwide survey of practices and form content

**DOI:** 10.1111/vox.70104

**Published:** 2025-08-25

**Authors:** Viviane Pereira, Denise Brunetta, Rosana Cipolotti

**Affiliations:** ^1^ Faculty of Medicine Federal University of Cariri Juazeiro do Norte Ceará Brazil; ^2^ Division of Transfusion Medicine (HEMOCE) Centro de Hematologia e Hemoterapia do Ceará – HEMOCE Fortaleza Ceará Brazil; ^3^ Division of Transfusion Medicine (EBSERH) Empresa Brasileira de Serviços Hospitalares – EBSERH Fortaleza Ceará Brazil; ^4^ Health Sciences Graduate Program Federal University of Sergipe Aracaju Sergipe Brazil

**Keywords:** blood transfusion, consent form, patient safety

## Abstract

**Background and Objectives:**

Obtaining consent for blood transfusion is a critical component of patient safety and promotes active patient engagement. This study aimed to assess how this practice is currently implemented in Brazil.

**Materials and Methods:**

A cross‐sectional study was conducted from September 2024 to January 2025. Healthcare professionals involved in transfusion procedures across Brazil were invited to complete an electronic questionnaire based on international recommendations. Data were analysed using descriptive statistics.

**Results:**

Of 109 responses, 9 were excluded due to duplication. In the final sample, 63% were professionals affiliated with teaching hospitals and 61% worked in services performing over 200 transfusions per month. Overall, 21% reported not using a consent form prior to transfusion. Among the remaining respondents, 15% stated that consent could be obtained at admission, regardless of transfusion indication. The validity period of consent and procedures for legally incapacitated patients varied widely. Additionally, 33% of forms did not mention benefits, 82% of them did not include a description of the transfusion process, only 20% addressed alternatives to transfusion, and 33% included an option for revocation.

**Conclusion:**

The transfusion consent process in Brazil requires significant improvement. Strengthening this practice is essential to uphold patient autonomy and enhance engagement of patients and families in healthcare decisions.

**Highlights**

- Significant inconsistencies were identified in transfusion consent practices across Brazil, with notable variation in content, validity periods and procedures, underscoring the need for standardization.
- A considerable number of transfusions occur without documented consent; and even when consent forms are used, essential information, such as alternatives and revocation rights, is frequently missing.
- Improving the transfusion consent process might be achieved through national guidance, standardized forms, professional training and local audits.

## INTRODUCTION

It is well established that no blood transfusion is entirely risk‐free. Although recent advances have mitigated certain transfusion‐related risks, others remain unavoidable. Nonetheless, a substantial number of transfusions are still performed without clear clinical indications, often based solely on laboratory values [1, 2].

In response, the World Health Organization (WHO) has promoted patient blood management (PBM) strategies, which emphasize patient‐centred care by optimizing red cell mass, minimizing blood loss and harnessing physiological tolerance of anaemia to reduce transfusion needs [3].

Within this patient‐centred framework, patient and family engagement, informed consent and shared decision‐making are key principles [3]. These elements are also reinforced in the WHO's Global Patient Safety Action Plan, which seeks to minimize preventable harm in healthcare worldwide [4].

Obtaining formal consent before transfusion is not mandatory in all countries, but its importance has grown in parallel with PBM adoption [5]. Well‐informed patients—aware of benefits, risks and alternatives—can participate more actively in treatment decisions and may refuse transfusion based on religious or personal beliefs. Beyond supporting patient autonomy, discussing the transfusion with the patient in advance helps them recognize and report adverse events more promptly [2].

Prominent international organizations, such as the Advisory Committee on the Safety of Blood, Tissues and Organs (SaBTO) and the American Association of Blood Banks (AABB), have defined minimum requirements for valid transfusion consent [6, 7]. However, many patients still undergo transfusions without fully understanding potential risks, benefits or alternatives [8].

While these guidelines are not formally adopted in Brazil, they are widely referenced in academic and professional settings. Notably, AABB accreditation has been implemented by several major transfusion centres in the country, promoting adherence to internationally recognized quality and safety standards.

In Brazil, approximately 3 million blood component transfusions are performed annually [9]. Although informed consent is mandatory for blood donation in the country, no such requirement currently exists for receiving transfusions [10]. Given the growing adoption of PBM practices nationwide, it is imperative to broaden the discussion on this issue.

This study investigates how transfusion consent is currently implemented across Brazil, based on the perspectives of professionals working in transfusion services.

## MATERIALS AND METHODS

This cross‐sectional observational study surveyed healthcare professionals from all regions of Brazil to assess how the informed consent process for blood transfusions is implemented. Geographical regions were categorized according to the official divisions defined by the Brazilian Institute of Geography and Statistics (IBGE).

An electronic questionnaire, developed based on the SaBTO and AABB guidelines [6, 7], was used to evaluate the essential elements included in transfusion consent forms. The instrument consisted of 28 questions, divided into three sections: participant demographics, procedural aspects of consent and analysis of transfusion consent forms. The content validity of the instrument was ensured through critical review by three transfusion medicine experts prior to distribution.

The study targeted professionals affiliated with transfusion services and directly involved in inpatient or outpatient transfusion procedures. To avoid institutional bias, responses from professionals working at the same transfusion service were excluded. Participants were recruited through a snowball sampling technique, given the logistical challenges of accessing eligible professionals across Brazil's diverse geographic regions. The link to the survey was disseminated to potential participants through three channels: direct communication, social media platforms (such as WhatsApp groups) and email correspondence.

According to Brazilian Health Regulatory Agency (ANVISA), approximately 1600 transfusion services operate in Brazil, linked to both public and private institutions. The proportion of these services utilizing their own consent forms is unknown. This study aimed to analyse 100 responses concerning the consent process, ensuring representation from all major geographic regions of the country.

Data were collected between September 2024 and January 2025. Participation was voluntary and anonymous. All participants provided informed consent prior to completing the online survey. The study received approval from the Research Ethics Committee of the Federal University of Cariri (CAAE: 80803224.2.1001.5698). Data were compiled in Microsoft Excel and analysed using descriptive statistics.

## RESULTS

A total of 109 healthcare professionals participated in the survey. Nine responses were excluded to avoid institutional overrepresentation, ensuring that the practices of a single institution were not disproportionately reflected.

**FIGURE 1:**
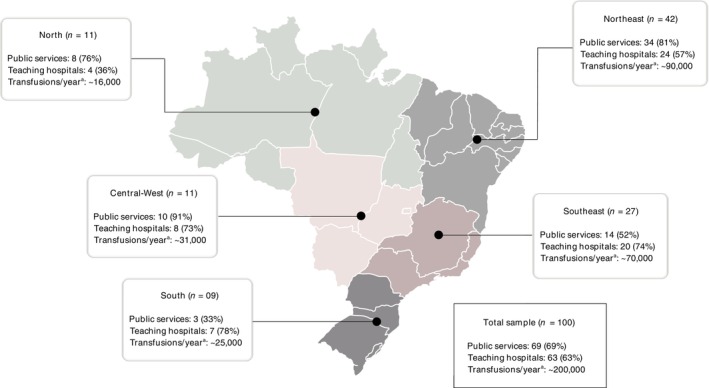
Distribution of survey responses and characteristics of the services represented. ^a^The number of transfusions per year indirectly analysed in each region was estimated based on the average monthly transfusions reported for the transfusion service indicated by the respondents.

The majority of respondents were affiliated with teaching hospitals (63%; 63/100) and worked in services performing more than 200 transfusions per month (61%; 61/100). The most represented regions were the Northeast (42%; 42/100) and Southeast (27%; 27/100), which correspond to the two regions with the highest number of hospital beds in Brazil. Additional sample characteristics are shown in Figure 1.

Twenty‐one per cent (21/100) of respondents reported not using a consent form prior to transfusion. Physicians were not always the professionals responsible for obtaining consent—this occurred in only 58% (46/79) of the cases. Excluding services that perform transfusions exclusively in outpatient settings, 15% of responses (11/74) indicated that consent could be obtained at hospital admission, irrespective of whether a transfusion had already been indicated.

In scenarios where the patient is unable to provide consent—emergency transfusions, or elective transfusions involving unconscious or legally incapacitated adults, or paediatric patients—a wide range of institutional protocols were reported (see Table 1).

**TABLE 1:** Practices for obtaining consent when patients are unable to provide it.

Who is authorized to provide consent for transfusion in	Number of responses	Proportion of responses (%)
1. Emergency transfusion (*n* = 90^a^)		
Doctor describes the reason for transfusion in medical records	31	35
The consent should be obtained later, as soon as possible	27	30
No specific conduct. The emergency is characterized by the patient's evolution in the medical record	12	13
The doctor must sign the form	10	11
The form includes a designated field to indicate that the situation is an emergency	8	9
Two doctors must sign the form	1	1
Unknown conduct	1	1
2. Elective transfusion in unconscious/incapable adults (*n* = 90^b^)		
Any legal guardian of the patient	41	46
Any companion present at the institution	17	19
Family member of any degree	10	11
First‐degree relatives	09	10
There is no formal definition by the institution	9	10
The assistant doctor	4	4
3. Elective transfusion in paediatric patient (*n* = 78^c^)		
Parents (biological or adoptive)	35	45
Any companion present at the institution	13	17
There is no formal definition by the institution	8	10
Any legal guardian of the patient	7	9
First‐degree relatives	7	9
Family member of any degree	7	9
The assistant doctor	1	1

^a^ The number of answers varied among questions because it was possible to include more than one option.

^b^ Three professionals who work exclusively with paediatric transfusions were excluded from this analysis.

^c^ Four professionals not involved in paediatric transfusions were excluded from the analysis.

Regarding the validity period of the consent form for hospitalized patients, 58% (43/74) indicated that the form covered the entire length of stay. In contrast, 28% (21/74) required new consent for each transfusion, and 11% (8/74) reported institutionally defined validity periods ranging from 1 to 12 months.

Regarding content, only 43% (34/79) of respondents indicated that the clinical indication for transfusion was included, while 66% (52/79) reported that the benefits were addressed. Although most forms included potential transfusion risks (95%—74/79), only 80% (59/74) described the symptoms associated with those risks. The least frequently included items were a description of the transfusion process itself (18%—14/79) and information on temporary restrictions related to blood donation after transfusion (9%—7/79).

Regarding the patient's right to refuse a transfusion, 67% (53/79) of the forms included this option. However, only 20% (16/79) mentioned alternatives to transfusion, and just 33% (26/79) included an option to revoke previously given authorization.

## DISCUSSION

The requirement to obtain consent prior to blood transfusion is a relatively recent practice. While well regulated in some high‐income countries, it remains insufficiently addressed in many others, where medical practice is still predominantly paternalistic, in which patients are expected to comply with physicians' decisions [11].

To our knowledge, this is likely the first study to examine transfusion consent practices across Brazil. The findings reveal a lack of standardization regarding content, validity periods and procedures used to obtain consent.

Based on the transfusion volumes reported by respondents working in services that do not use written consent forms, we estimate that at least 50,000 transfusions per month may occur in Brazil without formal documentation of consent. While this does not necessarily imply the absence of discussion, such interactions may go undocumented. Although not explicitly required by international guidelines, written consent forms serve as valuable tools to formalize the process, helping to document informed discussions and to support effective communication, transparency and patient engagement.

The sample was composed predominantly of professionals from large teaching hospitals, suggesting that many healthcare workers are trained in environments where transfusion consent is not consistently emphasized.

Physician involvement in the consent process appeared inconsistent, despite their ethical and clinical responsibilities to prescribe transfusions and assess individual risks and benefits. Obtaining transfusion consent at admission, often without reference to a specific indication, and extending its validity over prolonged periods raises significant concerns. Although this approach may be administratively convenient, it does not account for the fact that each transfusion decision involves a distinct clinical context, with specific risks, benefits and available alternatives. Moreover, a patient's preferences may change over time. In line with the principles of shared decision‐making, transfusion consent should ideally be obtained close to the time of the proposed intervention, after a discussion tailored to the specific situation and conducted by the prescribing clinician.

With respect to the content, only one participant reported using a form fully aligned with SaBTO and AABB recommendations. Potential transfusion risks were the most frequently reported topic; however, not all consent forms mentioned the symptoms related to these risks, and only a small percentage included a description of the transfusion process itself.

Given that transfusion is not without risks, some of which can be life‐threatening, it is essential that all patients for whom blood components are indicated receive adequate information about the benefits, risks and available alternatives [6, 7]. Moreover, patients must be made aware of their right to refuse treatment. Only with this information can a truly shared decision be made.

Many transfusion‐related adverse events result from preventable errors, including patient misidentification. While most transfusion reactions are mild, some can be life‐threatening and require prompt recognition and immediate clinical intervention [1]. Beyond supporting shared decision‐making, obtaining informed consent may strengthen haemovigilance activities. Educating patients about the steps involved in a safe transfusion and the symptoms of potential adverse reactions is essential for the early detection of complications and the prevention of errors [2].

The absence of a robust and standardized consent process suggests a gap that must be addressed through increased institutional support for PBM practices across Brazil. Healthcare professionals involved in transfusions must be proficient in the indications for each blood component, which go beyond simple laboratory thresholds [2]. All physicians should understand the risks associated with blood transfusions and seek to avoid them whenever possible. They should also be prepared to manage patients who refuse transfusion, supported by institutional protocols outlining available alternatives.

This study has several limitations. Data were derived from self‐reported information, as consent forms were not directly examined. The use of a non‐probabilistic snowball sampling method limits generalizability. Smaller institutions were underrepresented. Although ethical and safety standards apply irrespective of institutional size, such settings may face distinct operational challenges. Future research should explore how consent is obtained in services without written forms, whether local protocols exist, how provider–patient interactions are conducted, and whether any documentation is used. The perspectives of patients and families also warrant further investigation.

Ensuring transfusion safety requires the active engagement of all stakeholders, including patients and their families. The present findings underscore two critical concerns. First, a substantial proportion of transfusions in Brazil appear to occur without a written consent form. Although verbal discussions may take place, unless these are carefully documented in the patient's medical notes, the lack of recorded evidence limits transparency and accountability. Second, the use of consent forms is marked by significant variability in both implementation and content, potentially compromising the quality of informed and shared decision‐making.

According to WHO's PBM implementation guide, patient empowerment is critically dependent on transparent communication of risks, benefits, alternatives and potential complications prior to medical procedures, including transfusions. However, the WHO highlights that pro forma consent procedures, often developed locally and delegated to non‐prescribing staff, may fail to ensure meaningful patient participation in transfusion decisions. WHO advocates for a legally mandated and standardized consent process aligned with PBM principles [12]. Our findings reinforce this recommendation and underscore the need for national strategies in Brazil to strengthen the ethical, clinical and educational foundations of transfusion consent.

Improving transfusion consent practices requires coordinated action across regulatory, institutional and educational domains. The development of national guidelines is essential to define the core components of informed consent, clarify when and how it should be obtained, and establish minimum documentation standards. Training programmes for healthcare professionals on transfusion ethics and communication, along with educational resources for patients and families, may strengthen shared decision‐making and patient engagement.

In alignment with national healthcare policies in Brazil, these strategies could be integrated into Brazil's National Patient Safety Program (PNSP) [13], which provides an institutional framework through mandatory safety committees and national protocols, such as safe identification, infection prevention, safe surgery and medication safety. Incorporating transfusion consent within this structure would enhance consistency, documentation and patient‐centred communication. Moreover, audit and surveillance mechanisms already established under the PNSP could support monitoring and continuous improvement.

We hope these recommendations will contribute to strengthening transfusion consent practices in Brazil and offer relevant insights for other countries facing similar challenges.

## CONFLICT OF INTEREST STATEMENT

The authors declare no conflict of interest.

## Data Availability

The data that support the findings of this study are available from the corresponding author upon reasonable request.
